# Incidence of Urethral Stricture in Patients with Adult Acquired Buried Penis

**DOI:** 10.1155/2017/7056173

**Published:** 2017-03-30

**Authors:** Aron Liaw, Lanette Rickborn, Christopher McClung

**Affiliations:** The Ohio State University, Columbus, OH, USA

## Abstract

*Introduction*. Concealed-buried penis is an acquired condition associated with obesity, challenging to both manage and repair. Urethral stricture is a more common disorder with multiple etiologies. Lichen sclerosus is a significant known cause of urethral stricture, implicated in up to 30%. We hypothesize that patients with buried penis have a higher rate of urethral stricture and lichen sclerosus than the general population.* Methods*. We retrospectively reviewed a single surgeon's (CM) case logs for patients presenting with a buried penis. All patients were evaluated for urethral stricture with cystoscopy or retrograde urethrogram either prior to or at the time of repair for buried penis. Those that had surgical repair or biopsy were reviewed for presence of lichen sclerosus.* Results*. 39 patients met inclusion criteria. Of these, 13 (33%) had associated stricture disease. The location of the strictures was bulbar urethra (38%), penile urethra (15%), and meatus or fossa navicularis (62%). Five patients had lichen sclerosus and urethral stricture disease, while 3 had lichen sclerosus without stricture. 11/13 stricture patients were treated. Six underwent dilation, 3 underwent meatotomy, and 2 underwent urethroplasty. No significant recurrences of stricture were seen.* Conclusion*. Patients with a concealed penis are more likely than the general population to have a urethral stricture and/or LS. Patients presenting with concealed penis should also be evaluated for a urethral stricture.

## 1. Introduction 

Obesity rates are rising in the United States. 34.9% of adults and 17% of children (ages 2–19) are now classified as obese [1]. Buried penis in adults is generally an acquired condition [2] and has a known association with obesity [3]. The term concealed penis refers to a spectrum of disorders in which the penis is not protuberant due to a variety of causes including obesity. Higuchi et al. classify adult concealed penis into the following categories: a trapped penis caused by genital skin scarring, a buried penis caused by obesity, and complex buried penis which arises from a combination of the two aforementioned factors. Due to the rising obesity in the United States, the buried penis prevalence rates are likely to increase. Patients with acquired buried penis frequently have multiple comorbidities, both urologic and nonurologic [2]. The challenges presented by these patients are considerable, and a number of studies and reports have looked at the surgical methods of repair [2–4].

Urethral stricture disease occurs with a prevalence of 229–627 per 100,000 males [5]. Iatrogenic and idiopathic causes of urethral stricture are the most common etiologies. Lichen sclerosus, an inflammatory condition, is also a known cause of urethral stricture and has been cited to be responsible for up to 30% of urethral stricture [6–8]. Lichen sclerosus is known to be associated with buried penis, in both children [9] and adults [10]. We hypothesize that patients with a buried penis have urethral stricture disease and lichen sclerosus more commonly than reported in the general population.

## 2. Methods 

After institutional review board approval (IRB #2014H0326), we retrospectively reviewed a single surgeon's (CM) case logs for patients presenting for evaluation of a concealed penis. Queries were made for a diagnosis code of ICD-9 code 752.65 (buried penis). BMI values were tabulated. It was the practitioner's practice pattern to evaluate all patients for a urethral stricture prior to or at the time of surgical repair of a concealed penis, in the form of either a cystoscopy or retrograde urethrogram. For patients meeting inclusion criteria, charts were reviewed to determine the number of patients with a urethral stricture, as well as the location of the stricture. Of those patients who had surgical intervention for their concealed penis, pathology records were reviewed to determine the number of patients with pathological evidence of lichen sclerosus.

## 3. Results 

There were a total of 39 patients that met inclusion criteria. Of the 39 patients, 13 (33%) had associated stricture disease (Table 1). Of these 13 patients, the location of the strictures was bulbar urethra in 5 (38%), mid to proximal penile urethra in 2 (15%), and meatus or fossa navicularis in 8 (62%) (Table 2).

Of the 13 patients with urethral stricture disease, 11 received treatment. The 2 that did not receive treatment were also individuals that elected not to undergo treatment of their buried penis, usually due to other comorbidities. For those that did receive treatment 6/11 (55%) had dilatation, 3/11 (27%) had meatotomies, and 2/11 (18%) underwent urethroplasty. Those who had a dilation were offered urethroplasty prior to endoscopic intervention.

At a median follow-up of 12 months, one patient was found to have a nonobstructive annulation (>16 Fr) at his urethroplasty site but was asymptomatic. No other failures or recurrences of stricture disease were found.

The average BMI was 49.77 in patients with stricture disease and was 45.73 in those without stricture disease. This difference was not found to be statistically significant (*p* = 0.249).

Of the 39 patients in this series of concealed penis, 14 had surgical intervention. Three of these were treated with a palliative dorsal slit, and the rest were treated with formal reconstruction. Formal reconstruction required mons resection, resection of any cicatrix site when present, and split thickness skin graft to the penis. Pathology was available in all cases. Patients who had a dorsal slit had an excisional biopsy of the concealment site at the time of the procedure. Eight of the 14 (57%) demonstrated lichen sclerosus on final pathology.

There were 5 patients that had lichen sclerosus and stricture disease, and 8 patients that had lichen sclerosus overall. In this cohort, those that had lichen sclerosus were at increased risk of having urethral stricture disease (RR 2.42, 95% CI 1.09–5.41). The average BMI was 51.63 in patients with lichen sclerosus, and 46.33 in those without (−10.658–2.878 95% CI). However, this was not shown to be statistically significant (*p* = 0.147).

## 4. Discussion 

As obesity rates in the US continue to rise [1], the prevalence of concealed penis can also be expected to rise. This condition can lead to a number of urologic comorbidities. Donatucci and Ritter [11] describe symptoms of chronic urinary soiling, painful urination, inability to achieve penetration, or painful erection in most to all patients with obesity-induced concealed penis. In extreme cases, significant prepubic phlegmon and urinary retention can occur [12].

The prominent suprapubic fat pad that leads to concealed penis creates an environment conducive to bacterial and fungal growth and skin breakdown. It is thought that this environment leads to an inflammatory cycle that predisposes to cicatricial scar and lichen sclerosus. Lichen sclerosus is indeed a common finding in patients with buried penis. In our series, almost 50% of patients had some element of lichen sclerosus, similar to previous series.

Lichen sclerosus is known to carry a significant risk increase for urethral stricture [13] and can affect any area of the anterior urethra [14]. Palminteri et al. found lichen sclerosus to be a major cause of long urethral and penile strictures [14], and it is suggested that distal obstruction such as that from lichen sclerosus or meatal stenosis leads to high pressure voiding and inflammation of the periurethral glands, causing progression of stricture disease [15, 16]. This is similar to the Koebner phenomenon, which is thought to be a factor in the formation of lichen sclerosus in the phimotic penis [17, 18].

In this light, it is perhaps not surprising that we find an association between concealed-buried penis and urethral stricture. Similarly, the fact that the majority of strictures found were at the meatus or fossa navicularis is also not unexpected given the proinflammatory nature of the local environment at the glans. However, there is also a high rate of penile and bulbar stricture in this cohort, and 3 of the 5 patients that had lichen sclerosus also had more proximal strictures, including one patient with multiple discrete strictures and another who had stricture of essentially the entire penile urethra.

Even in those patients who did not have biopsy that demonstrated clinical lichen sclerosus or chronic inflammation, however, there was still a very high prevalence of stricture disease. Five patients had no evidence of chronic inflammation on their pathology but nonetheless had stricture disease.

No other significant associations were identified, though the cohort is small. Average BMI was not significantly different in patients with strictures than those without. All patients in this study are adults. Buried penis in children is a more common condition, but there is no literature suggesting any association with urethral stricture disease.

The main limitation of this study is the small sample size and the retrospective nature of the design. Short follow-up is also a limitation and is likely the reason of lack of recurrence in men treated with dilation alone. To our knowledge, this is the first study to demonstrate an increased incidence of urethral stricture disease in patients with a concealed penis relative to the general population. Due to this finding, it has become our common practice to investigate all buried penis patients for urethral stricture and to treat it where appropriate separately from their buried penis repair.

## 5. Conclusion

Patients with a concealed penis are more likely than the general population to have a urethral stricture and are more likely to have LS. Patients presenting for evaluation of a concealed penis should also be evaluated for a urethral stricture disease.

## Figures and Tables

**Table 1:** 

Total patients	39
Urethral stricture	13 (33%)
Lichen sclerosus	8 (20%)
Lichen sclerosus + urethral stricture	5 (12.8%)

**Table 2:** Patients with urethral stricture.

Age	BMI	Location of stricture	Length of stricture	Treatment of stricture	Months of follow-up
35	63	Penile urethra	>5 cm	Dilation	1
43	57	Meatus	<1 cm	Meatotomy	1
35	47	Meatus + proximal bulbar	1 cm at bulb	Meatotomy only	5
48	39	Fossa navicularis	1 cm	None	12
24	34	Meatus	<1 cm	None	0
54	39	Meatus	<1 cm	Dilation	17
65	46	Penile urethra	>5 cm	Dilation	14
42	53	Meatus	1 cm	Meatotomy	1
50	36	Meatus	<1 cm	Urethroplasty	24
55	57	Fossa navicularis + bulbar urethra	1 cm + 1 cm	Dilation	33
55	73	Bulbar urethra	2 cm	Dilation	6
59	42	Bulbar urethra	5 cm	Posterior urethroplasty	24
40	63	Bulbar urethra	3 cm	Dilation	43
